# ESPO and Biological Sciences Section Symposium: Exploring New Horizons in Geroscience

**DOI:** 10.1093/geroni/igaf122.1924

**Published:** 2025-12-31

**Authors:** Nisi Jiang, Sarah Ashiqueali

## Abstract

Over the past year, the field of gerontology has made significant strides, deepening our understanding of the biological mechanisms underlying aging and paving the way for potential interventions. Breakthroughs in cellular and molecular research have provided new insights into the fundamental drivers of aging, while emerging therapeutic approaches show promise in extending healthspan. Advances in genetics, epigenetics, and metabolic regulation are shaping novel strategies to slow down or even reverse aspects of aging. Concurrently, clinical studies are actively evaluating novel interventions that could translate these scientific discoveries into real-world applications. Join us to explore the latest frontiers in aging research, gain insights from leading experts, and connect with the next generation of scientists driving innovation in this rapidly evolving field.

